# Cell Lineage Identification and Stem Cell Culture in a Porcine Model for the Study of Intestinal Epithelial Regeneration

**DOI:** 10.1371/journal.pone.0066465

**Published:** 2013-06-28

**Authors:** Liara M. Gonzalez, Ian Williamson, Jorge A. Piedrahita, Anthony T. Blikslager, Scott T. Magness

**Affiliations:** 1 Center for Comparative Medicine and Translational Research, North Carolina State University, Raleigh, North Carolina, United States of America; 2 Department of Clinical Sciences, North Carolina State University, Raleigh, North Carolina, United States of America; 3 Molecular Biomedical Sciences, North Carolina State University, Raleigh, North Carolina, United States of America; 4 Department of Medicine, University of North Carolina, Chapel Hill, North Carolina, United States of America; 5 Cell Biology & Physiology, University of North Carolina, Chapel Hill, North Carolina, United States of America; 6 UNC/NCSU Biomedical Engineering, University of North Carolina, Chapel Hill, North Carolina, United States of America; National Cancer Institute, United States of America

## Abstract

Significant advances in intestinal stem cell biology have been made in murine models; however, anatomical and physiological differences between mice and humans limit mice as a translational model for stem cell based research. The pig has been an effective translational model, and represents a candidate species to study intestinal epithelial stem cell (IESC) driven regeneration. The lack of validated reagents and epithelial culture methods is an obstacle to investigating IESC driven regeneration in a pig model. In this study, antibodies against Epithelial Adhesion Molecule 1 (EpCAM) and Villin marked cells of epithelial origin. Antibodies against Proliferative Cell Nuclear Antigen (PCNA), Minichromosome Maintenance Complex 2 (MCM2), Bromodeoxyuridine (BrdU) and phosphorylated Histone H3 (pH3) distinguished proliferating cells at various stages of the cell cycle. SOX9, localized to the stem/progenitor cells zone, while HOPX was restricted to the +4/‘reserve’ stem cell zone. Immunostaining also identified major differentiated lineages. Goblet cells were identified by Mucin 2 (MUC2); enteroendocrine cells by Chromogranin A (CGA), Gastrin and Somatostatin; and absorptive enterocytes by carbonic anhydrase II (CAII) and sucrase isomaltase (SIM). Transmission electron microscopy demonstrated morphologic and sub-cellular characteristics of stem cell and differentiated intestinal epithelial cell types. Quantitative PCR gene expression analysis enabled identification of stem/progenitor cells, post mitotic cell lineages, and important growth and differentiation pathways. Additionally, a method for long-term culture of porcine crypts was developed. Biomarker characterization and development of IESC culture in the porcine model represents a foundation for translational studies of IESC-driven regeneration of the intestinal epithelium in physiology and disease.

## Introduction

Complete physiologic renewal of the intestinal epithelium occurs in approximately one week and is driven by a pool of IESCs at the crypt base [1]. This impressive rate of renewal is tightly controlled in homeostasis. Dysregulation of IESC renewal results in intestinal disorders such as small intestinal and colorectal cancer, which is the leading cause of digestive disease-related mortality [2], [3]. Impaired epithelial renewal can lead to ulceration, chronic inflammatory responses and sepsis [4], [5]. Since the description of IESCs in 1974 by Cheng and Lebond, investigators have attempted to understand the factors that control IESC-driven epithelial regeneration in physiology and disease [6].

In general, logistical and ethical issues minimize the use of humans or human- derived tissues for research and discovery pertaining to conditions of the intestinal epithelium. These obstacles highlight the need for a research model that closely mimics human intestinal anatomy, physiology, disease and injury processes. Currently, the vast majority of basic studies focused on intestinal epithelial diseases, injury and regeneration utilize rodent models. Rats and mice in particular represent an important, cost effective animal model for basic genetic, cellular and molecular biology of IESC-driven regeneration of the intestinal epithelium. Despite these advantages, significant differences between rodents and humans confound or prohibit translational studies [7].

Important anatomical, behavioral and environmental conditions that impact epithelial regeneration are more closely shared between pigs and humans than between mice and humans [8], [9]. Pigs and humans share parallel mucosal barrier physiology, food intake, enteric microbiota composition, and pathogenicity of key disease causing microbes [7]. Pigs, like humans, are true omnivores and share similar metabolic and intestinal physiologic processes [7], [9]. A mucosal *in vitro* permeability study demonstrated greater correlation between humans and pigs when compared to rats [8]. Importantly, it has been demonstrated that pigs represents a more physiologically relevant model of neonatal necrotizing enterocolitis, intestinal ischemia-reperfusion injury, acute mesenteric ischemia, short bowel syndrome, AIDS-associated opportunistic *Cryptosporidium* infection, and stress-induced intestinal dysfunction [10]–[22]. Additionally, a large animal model is likely to serve as a more physiological relevant model to study segmental assessment of radiation exposure, focally induced ischemia and reperfusion as well as transplantation and cell-based therapies.

Severe intestinal disease necessitates approximately 200 intestinal transplantations each year in the United States [2]. In a prospective cross-sectional study of patients, 40% of visceral allograft recipients died within 5 years of transplantation [23]. The impact of digestive disease on rates of mortality and morbidity as well as health care costs in the United States has created an urgent need for advances in transplantation and tissue replacement therapies [2]. A key factor to the success of many translational studies is the gross size of the animal model. The small size of the intestines of experimental rodent models often prohibits tissue manipulation or implementation of candidate surgical interventions such as tissue engraftment or transplantation. These limitations further highlight the need for a large animal model to advance cell or tissue based therapies.

This study focuses on eliminating many of the obstacles that limit the pig as a translational model to study IESC-driven regeneration of the intestinal epithelium. This work thoroughly characterizes the porcine intestinal mucosa by identifying, developing and validating a comprehensive set of reagents to study porcine stem/progenitor cells and their principal post-mitotic cell descendants *in situ* and in culture.

## Materials and Methods

### Ethics Statement

All animal studies were approved by the Institutional Animal Care and use Committee at North Carolina State University.

### Animals and sample collection

Tissues were obtained from healthy 6–8 week-old wild type Yorkshire cross pigs euthanized for reasons unrelated to this project. Sections from the gastrointestinal tract including the duodenum, jejunum, ileum, proximal and distal colon were sharply dissected.

### Histological and Immunofluorescence Analyses

Tissues were rinsed with 1× phosphate-buffered saline (PBS) and opened longitudinally along the anti-mesenteric boarder. For immunohistochemical analysis, tissue was fixed in 10% formalin, embedded in paraffin and sectioned (∼5–8 μm thickness). Slides were stained with hematoxylin and eosin to visualize crypt and villus morphology. For immunofluorescence, rinsed tissue was fixed in 4% paraformaldehyde (PFA) solution for 14–18 hours at 4°C. The tissue was transferred to 30% sucrose solution for at least 24 hours at 4°C, embedded in optimal cutting temperature (OCT) media, frozen and sectioned at 5–8 μm thickness using a cryotome and mounted on positively charged glass slides. Sections were washed three times with PBS to remove OCT. When necessary, heat induced epitope retrieval (HIER) was then performed by placing slides into reveal decloaker solution (Biocare Medical, Concord, CA) for 30 seconds at 120°C and then 90°C for 10 seconds, in a pressure cooker. The slides were allowed to cool at room temperature for 20 min prior to continuing. Tissue permeabilization was performed on all slides with PBS-0.3% Triton X-100 for 20 min, washed twice with PBS and incubated in blocking medium (Dako, Carpinteria, CA). Primary antibodies were applied to the tissue section in an antibody diluent (Dako) and incubated overnight at 4°C. Dilutions for functional antibodies were as follows: αSOX9 (rabbit, 1∶1000, Chemicon/Millipore, Temecula, CA), αMucin2 (rabbit; 1∶1000, Santa Cruz Biotechnology, Santa Cruz, CA), αLectin from Ulex europaeus-Atto 488 conjugate (1∶500, Sigma-Aldrich, St. Louis, MO), αsucrase isomaltase (goat, 1∶500, Santa Cruz Biotechnology), αCD326/EpCAM (rat, 1∶500, BioLegend, San Diego, CA), αVillin (goat, 1∶500, Santa Cruz Biotechnology), αCleaved Caspase 3 (rabbit, 1∶400, Cell Signaling Technology, Inc., Danvers, MA), αGlucagon (rabbit, 1∶250, Santa Cruz Biotechnology), αSomatostatin (goat, 1∶500, Santa Cruz Biotechnology), αCarbonic Anhydrase (goat, 1∶250, Santa Cruz Biotechnology), αProliferating cell nuclear antigen (mouse, 1∶100, Chemicon/Millipore), αSP-1 Chromogranin A (Bovine) (rabbit, 1∶1000, Immunostar, Hudson, WI), αSP-1 Chromogranin A (Porcine) (rabbit; 1∶1000, Immunostar), αBeta-catenin (mouse, 1∶200, Cell signaling technology, Inc.), αMinichromosome Maintenance Complex 2 (Goat, 1∶200, Santa Cruz Biotechnology) and αPhospho-histone H3 (rabbit, 1∶200, Cell Signaling technology, Inc.). The BrdU staining protocol was performed on donated tissue from animals treated as described using a monoclonal antibody against BrdU (mouse; 1∶100, Dako) [24]. All secondary antibodies (Jackson ImmunoResearch or Sigma, conjugated to Dylight 488, Cy 3 or Alexafluor 555) were diluted 1∶500, and counter stained with bisBenzimide H 33258 nuclear stain (1∶1000, Sigma). Background staining was negligible as determined by nonspecific IgG staining. Images were captured on an inverted fluorescence microscope (Olympus IX81, Tokyo, Japan) fitted with a digital camera (ORCA-flash 4.0 or -03G, Hamamatsu, Japan). The objective lenses used were X10, X20 and X40 with numerical apertures of 0.3, 0.45 and 0.6, respectively (LUC Plan FLN, Olympus, Tokyo, Japan). Immunohistochemically stained slides were imaged with an Olympus Bx45 microscope fitted with and Olympus DP72 camera. The objective lenses used were X10, X20 and X100 with numerical apertures of 0.3, 0.5 and 1.3 oil, respectively (UPlanFLN, Olympus).

Appropriate antibody specificity to cellular biomarker was supported by western blot analysis (Table 1), known localization from published immunofluorescence imaging of porcine [10], [24]–[27] and mouse studies [28]–[32], as well as consistent repeatability of staining and appropriate localization along the crypt villus axis.

**Table 1:** Functional/Cross Reactive Antibodies.

	Protein	Company		Catalog #	Host Species	Dilution 1° Atb	Antigen Retrieval	Functional for Western Blot/Band (KDa)
IESC								
	SOX9	Millipore		ab5535	rabbit	1:1000	Yes	Yes (56–65)
	HOPX	Santa Cruz		sc-30216	rabbit	1:500	Yes	
Proliferative								
	PCNA	Millipore		MAB424R	mouse	1:100	Yes	Yes (36)
Goblet								
	MUC2	Santa Cruz		sc-15334	rabbit	1:1000	No	
	UEA-1	Sigma		19337 Atto 488		1:500	No	
Enteroendocrine								
	CgA	Immunostar		20086 (Porcine)	rabbit	1:1000	Yes	Yes (75)
	CgA	Immunostar		20085 (Bovine)	rabbit	1:1000	No	
	Gastrin	Santa Cruz		sc-7783	Goat	1:250	Yes	
	Glucagon	Santa Cruz		sc-13091	rabbit	1:250	No	
	Somatostatin	Santa Cruz		sc-7819	Goat	1:500	No	
Absorptive Endocrine								
	Sucrase Isomaltase		Santa Cruz	sc-27603	Goat	1:500	Yes	Yes (200)
	CAII		Santa Cruz	sc-17244	Goat	1:250	No	Yes (29)
	CAII		Santa Cruz	sc-17246	Goat	1:500	Yes	
Epithelial								
	EpCAM		Biolegend	118212	mouse	1:500	No	
	Villin		Santa Cruz	sc-7672	Goat	1:500	Yes	
Apoptosis and Cell Cycle								
	Caspase 3		Cell Signaling	9661S	Rabbit	1:400	Yes	
	Phosph-histone H3		Cell Signaling	9701	Rabbit	1:200	Yes	
	Phospho-histone H3		Millipore	6–570	Rabbit	1:500	Yes	
	MCM2		Santa Cruz	Sc-9839	Goat	1:200	Yes	
	Beta catenin		Cell Signaling	2677	mouse	1:200	Yes	

### qRT-PCR

The jejunal mucosa was physically separated from seromuscular layers by scraping with a glass slide and was placed in RNAse free microtubes and immediately placed in liquid nitrogen. They were then stored at −80°C until use. Total RNA from jejunal tissue was extracted using the Qiagen RNeasy Minikit (Qiagen, Valencia, CA). Yield and quality of the extracts were determined by measuring absorbance at 260 and 280 nm (NanoDrop Technologies Thermo Fisher Scientific Wilmington, DE). The ratio of absorbance at 260∶280 was between 2.03 and 2.07. 1 µg of RNA was converted to cDNA using the iScript cDNA synthesis kit (Biorad) and pooled. The cycle conditions were 5 min at 25°C, cDNA synthesis at 42°C for 30 min, denaturation at 85°C for 5 min and held at 4°C. Primers were designed based on published sequences of the pig target genes either manually or using the NCBI online primer design tool (Primer-BLAST, http://www.ncbi.nlm.nih.gov/tools/primer-blast/), Primer3 input (version 0.4.0, frodo.wi.mit.edu/). The specificity of the primers was checked using the NCBI online Blast tool (Primer-BLAST, http://www.ncbi.nlm.nih.gov/tools/primer-blast/). Quantitative RT-PCR was performed utilizing the iTaq Universal SYBR green Supermix (BioRad). Standard curves were generated using serial dilutions of pooled cDNA from all three normal pigs tested in triplicate. The StepOnePlus Real time PCR system (Applied Biosystems by Life Technologies, Carlsbad, CA) was used. Cycle parameters included polymerase activation and DNA denaturation at 95°C for 30 sec. Forty cycles of amplification were performed with a 15 sec denaturation at 95°C and annealing/extension and plate read for 60 sec at 60°C. The melting curve analysis was performed at 65°C–95°C at 0.5°C increments, 5 sec per step. Melting curves were checked to ensure consistent amplification of a single PCR product. Primer efficiency was calculated using the equation, Efficiency  = 10^∧^(−1/slope) –1. All primer efficiencies were greater than 92%.

### Target validation by sequencing

RT-PCR amplicons were analyzed on a 1.5% Agarose TE gel to assess size. The amplicons were purified using the USB ExoSAP-IT PCR product cleanup reagent (Affymetrix, Santa Clara, CA). Samples were sequenced (GENEWIZ, Research Triangle Park, NC), and the sequences were aligned to the gene target using the NCBI online Blast tool and Vector NTI alignment program (Life Technologies) for validation.

### Transmission Electron Microscopy

TEM imaging was performed by the laboratory for advanced electron and light optic methods at North Carolina State University. The tissue fixation, preparation and image acquisition were performed as previously published [6], [33], [34].

### Crypt isolation, Enteroid Culture and Analysis

Tissue was taken from 2–14 day-old wild type Yorkshire piglets euthanized for other research purposes. An 8–10 cm segment of distal duodenum/proximal jejunum was surgically excised and opened longitudinally. The tissue was incubated for 20 min in a phosphate-buffered saline solution (PBS) containing 30 mM Ethylenediaminetetraacetic acid (EDTA), 10 µM Y-27632 (Selleck, Houston, TX), 1mM DTT (Sigma-Aldrich), and 100 µg/mL penicillin/streptomycin at 4°C on an orbital shaking platform moving at 80 rpm. Tissue was transferred into a 37°C pre-warmed PBS solution containing 30 mM EDTA, 10 µM Y-27632, and 100 µg/mL penicillin/streptomycin. The tissue was incubated in this solution at 37°C for 10 min and then shaken at 2.5 cycles sec-1 to mobilize the crypt/villi units. If the desired yield was not achieved, the tissue was incubated in solution at 37°C for an additional 2 min and shaken for an additional 30 sec up to 5 times. Longer incubations caused extremely poor survival. Following the final shake, the remnant intestine was removed from the solution and the crypt/villi units were quantified and pelleted.

The pelleted epithelium was re-suspended directly into hESC Matrigel (BD Bioscience, San Jose, CA) supplemented with 100 ngmL^−1^ recombinant mouse Noggin (Peprotech, Rocky Hill, NJ), 500 ngmL^−1^ recombinant human R-Spondin (R&D Systems, Minneapolis, MN), 50ngmL^−1^ recombinant mouse EGF (Life Technologies, Carlsbad, CA), 100 ngmL^−1^ recombinant human Wnt3a (R&D systems), 10 µM Y-27632 (Selleck), 10 µM SB202190 (Sigma), and 500 nM LY2157299 (Selleck). Between 50 and 200 crypt/villi units were plated in 50 uL of matrigel on a 48 well plate. After allowing the matrix to polymerize for 30 min at 37°C, each well was overlaid with 500 µL of Advanced DMEM/F12 containing the supplements 1× N-2 supplement (Life technologies), 1× B-27 supplement minus vitamin A (Life technologies), 1× Glutamax (Life Technologies), 100 µgmL^−1^ penicillin/streptomycin, 1 mM Hepes buffer (Life Technologies), and 1 mM N-Acetylcysteine. Growth factors were added to the media 48 hr after plating and every 72 hr following that. The entire volume of media was changed 72 hr following plating and every 72 hr after that. Every 1–2 weeks organoids were passaged at a 1∶5 ratio by mechanical dissociation, pelleting, and re-plating the pellet, into new Matrigel containing the growth supplements described above.

For histological studies, enteroids were fixed in a PBS solution containing 4% paraformaldehyde at room temperature for 20 min. Fixed enteroids were washed in a 30% sucrose solution and embedded in Optimal Cutting Temperature (OCT) media. Enteroids were cut into 5–8 µm sections and heat induced epitope retrieval was preformed when necessary by heating in reveal decloaker solution (Biocare Medical, Concord, CA) to 120°C for 30 sec and then 90°C for 10 sec inside a pressure cooker. Slides were allowed to cool to room temperature for 20 min prior to staining. Enteroids were permeabilized in a 0.3% Triton X-100 PBS solution for 20 min and then blocked in protein block solution (Dako, Carpinteria, CA) for 30 min. Primary antibodies, αSOX9, αMucin2, αsucrose isomaltase, and αPCNA, at the same dilutions previously described, were applied to the slides in antibody diluent (Dako) and incubated for 2 hr at room temperature. All secondary staining was preformed with Cy3 conjugated antibodies (Jackson ImmunoResearch, West Grove, PA) diluted 1∶500 in antibody diluent (Dako) incubated at room temperature for 45 min. Nuclei were marked with bisBenzimide H 33258 nuclear stain (Sigma/ Aldrich) diluted 1∶1000 in PBS and applied for 5 min at room temperature. Confocal images were obtained using a Zeis LSM 710 laser scanning microscope. The objective lenses used were X40 water and X63 oil with numerical apertures 1.1 and 1.4, respectively (C-Apochromat, Plan-Apochromat, Zeiss, Jena, Germany).

## Results

### Identification of cells of epithelial origin

**Figure 1:**
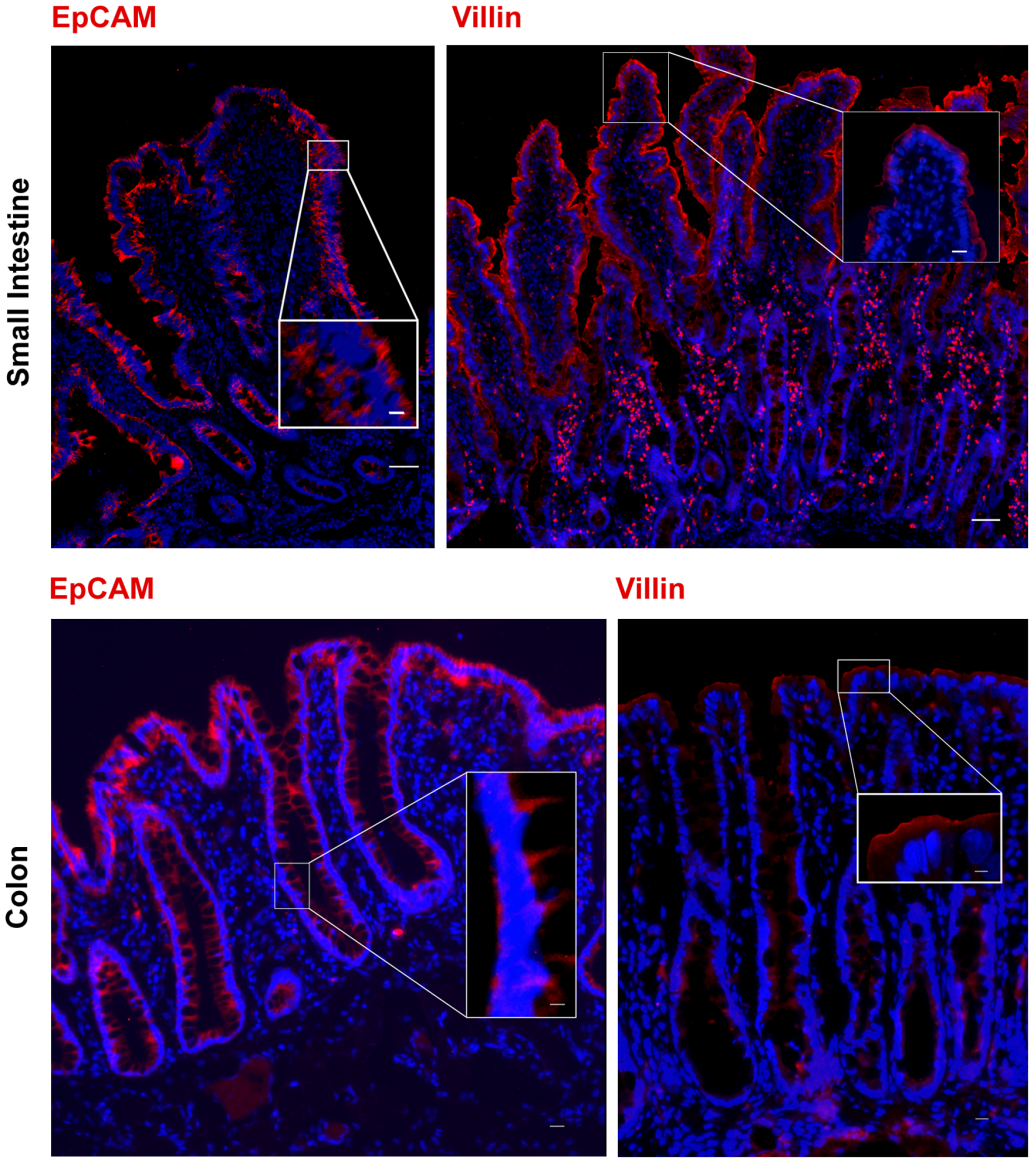
Markers to identify cells of epithelial origin. Identification of intestinal cells of epithelial origin within the porcine small intestine and colon are shown. Immunostaining for EpCAM, a pan-epithelial transmembrane protein, demonstrated expression in the basolateral membrane of all cells in both the small intestine and colon arranged along the luminal monolayer of the epithelial mucosa. Immunostaining for Villin, a protein associated with the microvillar actin filaments, showed a gradient of expression localized to the apical border of small intestinal and colonic epithelial cells with increasing intensity in cells located closer to the lumen. All specific markers (red). Nuclei (blue). Scale bar 200 µm, inset scale bar 50 µm.

To distinguish epithelial cells from those of mesenchymal and hematopoietic origin, we tested whether two candidate antibodies raised against mouse EpCAM (CD326) and human Villin would exclusively label pig epithelial cells. EpCAM is a pan-epithelial transmembrane protein that functions as a homotypic calcium-independent cell adhesion molecule [35]. EpCAM expression was observed in the basolateral membrane of all cells arranged along the luminal monolayer of the epithelial mucosa (Figure 1) [35]. In the small and large intestine, Villin expression is localized to the apical border of the intestinal epithelial cells due to its association with the microvillar actin filaments [36]. Villin expression demonstrated a gradient of increased staining intensity from the crypt toward the lumen as has been described in normal mammalian tissue (Figure 1) [36].

### Markers of proliferation and apoptosis identify cells within each stage of the cell cycle

**Figure 2:**
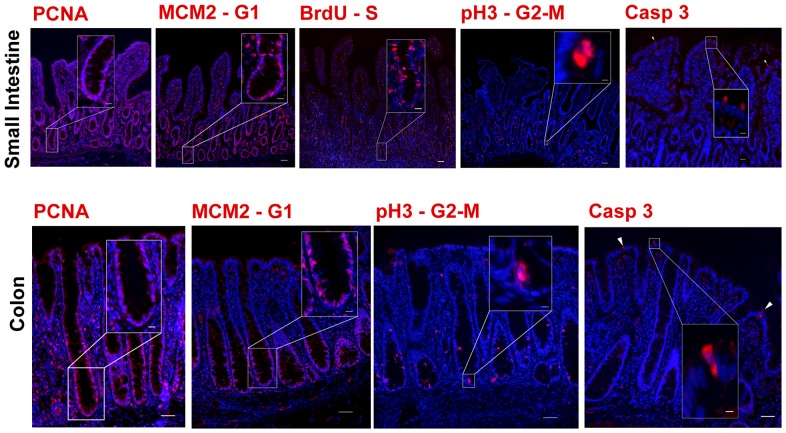
Markers to assess proliferation and apoptosis. Identification of proliferative cells in different stages of the cell cycle and those undergoing apoptosis in porcine small intestine and colon are shown. All proliferative markers localized to the nuclei of positive epithelial cells. Immunostaining for PCNA, a general marker for cellular proliferation, demonstrated the greatest number of positive cells compared to the other markers of proliferation. Immunostaining for MCM2, a marker of cells at the G1 stage of the cell cycle, was localized to a subpopulation of cells within the crypt base. Immunostaining for BrdU, a marker of cells within the S stage of the cell cycle, was also localized to a subpopulation of cells within the crypt base. Immunostaining for pH3, a marker for cells between the G2 – M stage of the cell cycle was similarly localized but to fewer cells. Immunostaining for cleaved caspase 3, an indicator of apoptosis, marked a few expressing cells near the villus tip within small intestine and the luminal surface of colon. All specific markers (red). Nuclei (blue) Scale bar 200 µm, inset scale bar 50 µm.

Assessing the proliferative capacity of IESCs and their progenitors is essential for monitoring regenerative responses in the small intestine and colon. Proliferating Cell Nuclear Antigen (PCNA) is accepted as a general proliferation marker and localized to the nucleus of the majority of cells constituting the crypt base in porcine tissue (Figure 2). Minichromosome Maintenance Complex 2 (MCM2) serves as a biomarker for cells that are peaking at G1-S phase [28]. The thymidine analogs BrdU or EdU are well established markers for cells in S-phase [29]. Both MCM2 and BrdU were localized within the nuclei of a subset of cells within the proliferative zone (Figure 2). Histone H3 is phosphorylated (pH3) at the end of prophase and represents a suitable marker for cells in G2-M-phase of the cell cycle. pH3 positive cells marked a minority of cells within all crypt bases consistent with the limited number of cells at this point in the cell cycle [30]. Immunostaining jejunal and colonic tissues for each of these proliferation markers demonstrates robust cross-reactivity with cells located in the proliferative zone of the crypt as has been demonstrated in mice (Figure 2) [28], [37], [38]. These validated antibodies for porcine gut tissue represent a comprehensive set of reagents for detailed study of the proliferative response in physiology, disease and injury induced regeneration.

Interrogating apoptotic dynamics is equally important to understanding mechanisms underlying a regenerative response [39]. Caspase3 cleavage represents the execution phase of apoptosis [39]. The antibody against cleaved human caspase 3 (CASP3) marked few cells at the villus tip in porcine small intestine and colon where an apoptotic event known as ‘anoikis’ typically occurs (Figure 2) [26]. Rare cells at the base of the crypts were observed which is consistent with rare apoptotic incidences in physiologic renewal.

### Identification of stem and progenitor cell populations

**Figure 3:**
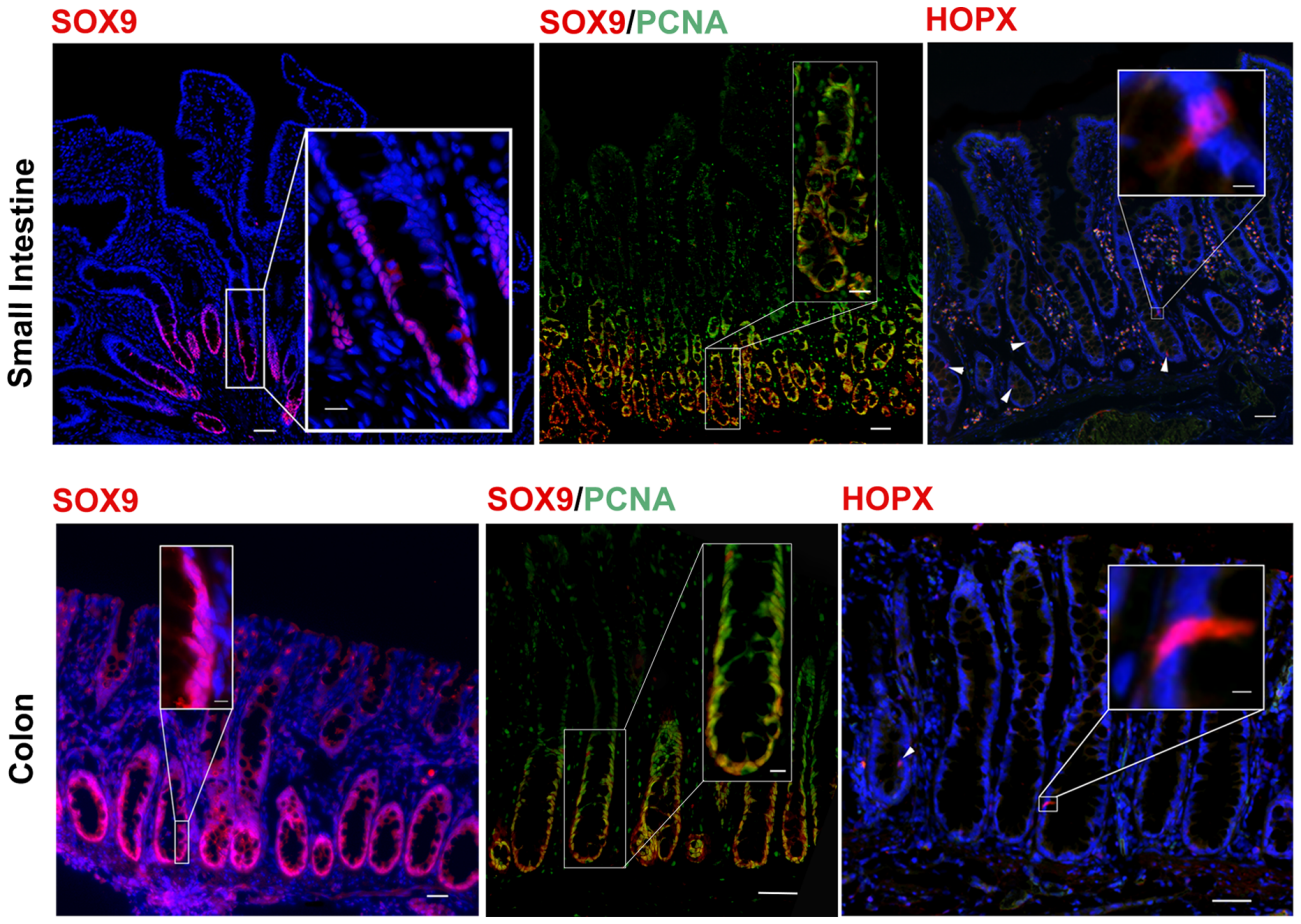
Markers to identify stem, progenitor, and transit amplifying cells. Identification of stem, progenitor, and transit amplifying cells in porcine small intestine and colon are shown. Immunostaining for SOX9, a member of the SRY-family of transcription factors that is primarily expressed in CBC cells and transit-amplifying progenitor cells, is localized to the nuclei of all cells within the crypt base of both the small intestine and colon. Immunostaining for SOX9 demonstrates colocalization with the proliferative cells (PCNA^+^) at the crypt base. Immunostaining for HOPX, an atypical homeodomain containing protein, demonstrated marking of cells consistent in location and numbers with a ‘reserve’ IESC population. All specific markers (red or green). Nuclei (blue). Scale bar 200 µm, inset scale bar 50 µm.

Next, we aimed to distinguish stem/progenitor cells from fully differentiated lineages. Recent evidence supports the presence of IESCs that exist in different states of proliferative capacity [40], [41]. Crypt-based columnar ‘active’ stem cells (CBCs) are located intercalated between Paneth cells in mice, are constantly dividing, and primarily responsible for the burden of homeostatic epithelial regeneration. Unfortunately, the commercially available antibodies used to detect the CBC population, LGR5, OLFM4, and CD24 did not demonstrate cross reactivity with active CBC stem cells in porcine intestinal tissue (data not shown; Table 2). However, antibodies raised against SOX9, a member of the SRY-family of transcription factors primarily expressed in CBCs and transit-amplifying progenitor cells, demonstrated the ability to detect proliferating cells in the base of the small intestine and colonic crypts with distinct localization to the nucleus of positive cells (Figure 3) [31], [42], [43].

**Table 2:** Non Functional Antibodies.

	Protein	Company	Catalog #	Host Species
IESC				
	LGR5	Santa Cruz	sc-68580	Goat
	LGR5	Origene	TA301323	Rabbit
	SOX4	Santa Cruz	sc-17326	Goat
	SOX17	Santa Cruz	sc-17355	Goat
	DCAMKL1	Abgent	AP7219b	Rabbit
	DCAMKL1	Abcam	37994	Rabbit
	MSI1	Millipore	AB5977	Rabbit
	OLFM4	Abcam	AB85046	Rabbit
	OLFM4/GC-1	Santa Cruz	Sc-84274	Rabbit
	CD24	BD Pharmingen	557436	Rat
	CD24	Thermoscientific	1279	Mouse
Progenitor				
	NEURO D	Santa Cruz	sc-1084	Goat
	HES1	Santa Cruz	sc-13844	Goat
	HES1	Millipore	D153-3	Rabbit
	NSUN1	Santa Cruz	sc-83439	Goat
	NOTCH1	Santa Cruz	sc-6014	Rabbit
Proliferative				
	Ki 67	Santa Cruz	sc-7846	Goat
	cMYC	Santa Cruz	sc-764	Rabbit
	Cyclin D1	Diagnostic Biosystems	RMAB003	Rabbit
Paneth				
	Lysozyme	Diagnostic Biosystems	RP028	Rabbit
	Lysozyme	Santa Cruz	sc-27598	Goat
	cKIT (CD117)	MBL	566	Rabbit
Enteroendocrine				
	Synaptophysin	Abcam	ab52636	Rabbit

Another putative stem cell population, termed ‘the +4 stem cells’, are a slower dividing ‘reserve’ or facultative stem cell that primarily reside above the Paneth cell compartment in mice and humans [41], [44]. In order to identify a putative +4 stem cell population, an antibody against human HOPX, an atypical homeodomain containing protein, was tested. Immunostaining for HOPX showed cross reactivity to cells in pig intestinal tissue with expression pattern restricted to the ‘+4’ stem cell zone consistent with what has been observed in mice (Figure 3) [32].

### Identification of absorptive cell lineage

**Figure 4:**
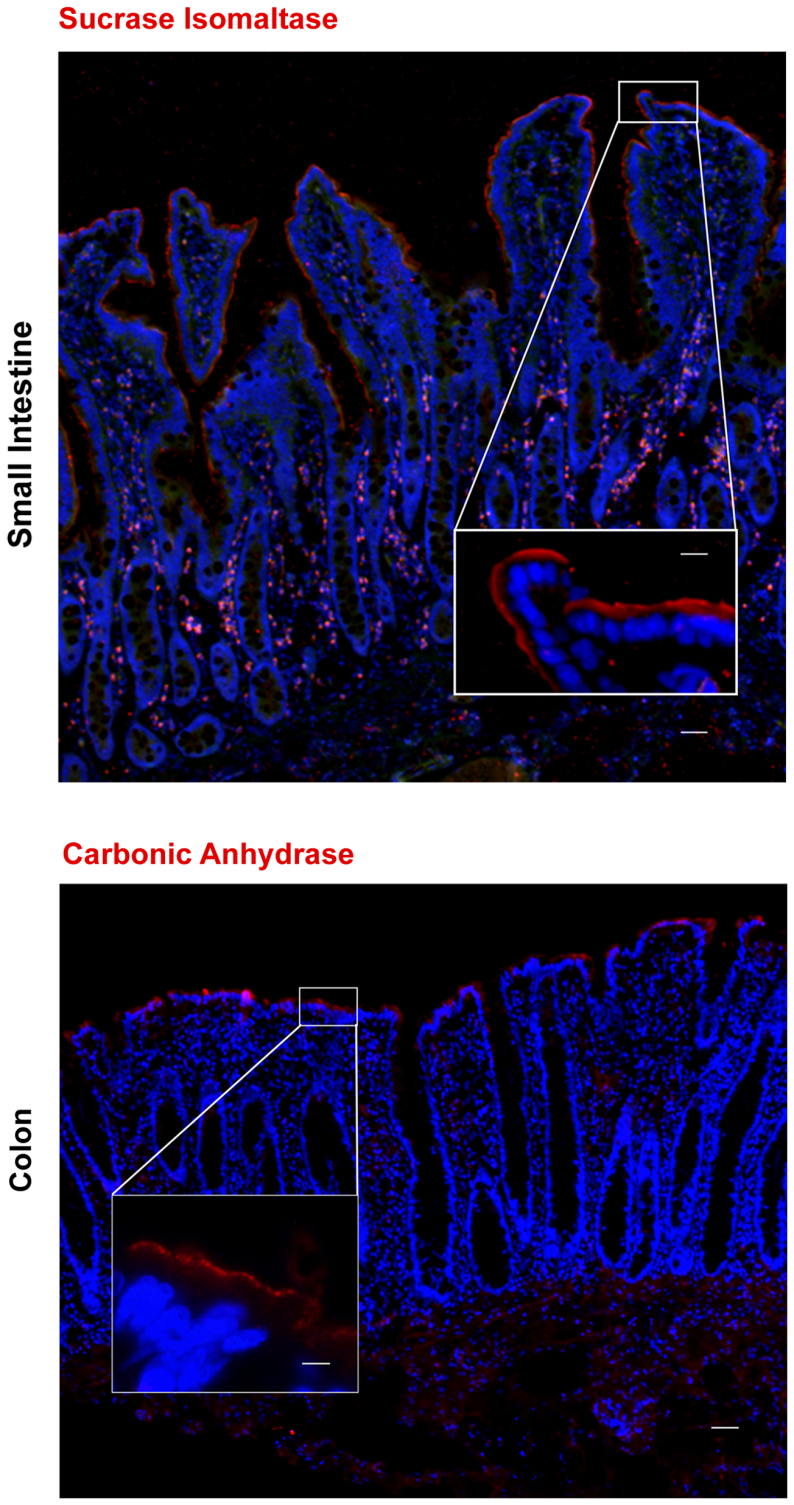
Markers to identify the absorptive cell lineage. Identification of absorptive enterocytes in porcine small intestine and colon are shown. Immunostaining for enterocytes demonstrates sucrase isomaltase, a digestive enzyme, marking the apical brush- border of cells in the small intestine. Carbonic anhydrase, a digestive enzyme, positively identified absorptive cells in the colon with staining localized to the apical brush- border of these cells. All specific markers (red). Nuclei (blue). Scale bar 200 µm, inset scale bar 50 µm.

Enterocytes function to absorb nutrients, electrolytes and water and are the predominant cell type within the intestinal mucosa [33]. The histologic identification of this cell lineage is used to assess whether appropriate cellular differentiation is occurring during a regenerative response [45], [46]. Immunostaining for the digestive enzymes, sucrase isomaltase (SIM) and carbonic anhydrase II (CAII) clearly demonstrates localization to the apical brush-border of the absorptive enterocytes in the small and large intestine, respectively. [47], [48]. There was no positive immunostaining of SIM in the colon or CAII in the small intestine indicating these antibodies are suitable to differentially distinguish between these two absorptive cell types (Figure 4).

### Identification of secretory cell lineage

**Figure 5:**
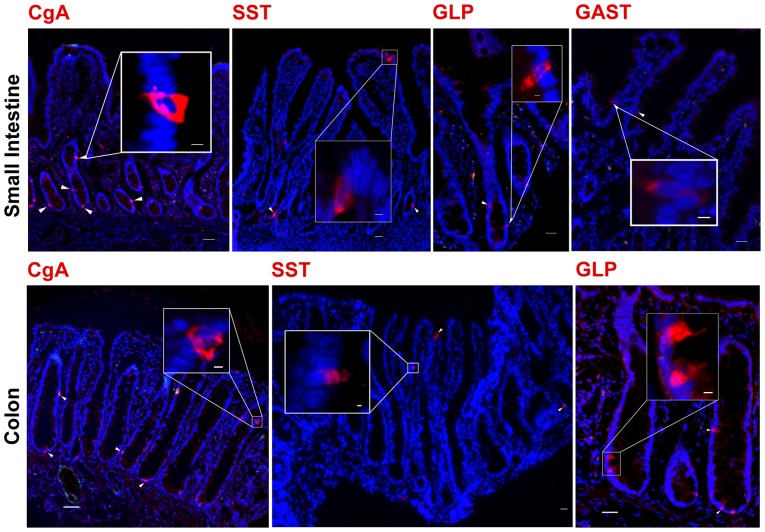
Markers to identify the secretory cell lineage. Identification of secretory cells in porcine small intestine and colon are shown. Immunostaining for enteroendocrine cells, the hormone secreting cells important to gut homeostasis, using antibodies against CgA, SST, GLP-1 and GAST, demonstrated staining within the cytoplasm of positive cells of the small intestine. A similar pattern of staining was identified in the colon for CgA, SST and GLP-1. All specific markers (red). Nuclei (blue). Scale bar 200 µm, inset scale bar 50 µm.

In mice and humans three primary secretory lineages exist, enteroendocrine cells, goblet cells and paneth cells. Enteroendocrine cells represent a minor population of cells that secrete various hormones that regulate gut physiology and appetite control [49]. Chromogranin A (CgA) is an acidic glycoprotein that localizes within secretory granules of nearly all enteroendocrine cells and is considered a general marker for all enteroendocrine cell subtypes [50]. Immunostaining for CgA localized to the cytoplasm of a minority of cells throughout the length of the small intestine and colon, consistent in morphology with enteroendocrine cells found in other animal species [31], [51] (Figure 5).

Subtypes of enteroendocrine cells could also be identified in pig intestinal epithelium. Gastrin (GAST) is a hormone secreted by G cells in the stomach and duodenum, and it functions as both a mucosal growth factor and stimulator of mast and parietal cells [49]. An antibody against human Gastrin (GAST) identified a minority of cells in the duodenum while no immunostaining was observed along other segments of the small or large intestine, an observation consistent with the expression pattern of GAST observed in humans [49] (Figure 5). Somatostatin (SST) is a hormone secreted by delta cells throughout the length of the intestine and functions to block the release of many gut hormones ultimately affecting epithelial transport and intestinal motility [49], [52]. SST-positive cells were observed intermittently in all segments of the porcine intestine (Figure 5). The main role of glucagon-like peptide 1 (GLP-1) is to delay gastric emptying and signal post prandial satiety and that of glucagon-like peptide 2 (GLP-2) is to stimulate mucosal enterocyte proliferation [53]. Reactivity to the glucagon antibody, with cytoplasmic localization, was observed within the enteroendocrine cells along the proximal distal axis of the small intestine (Figure 5) consistent with that observed in mice and humans [53], [54].

**Figure 6:**
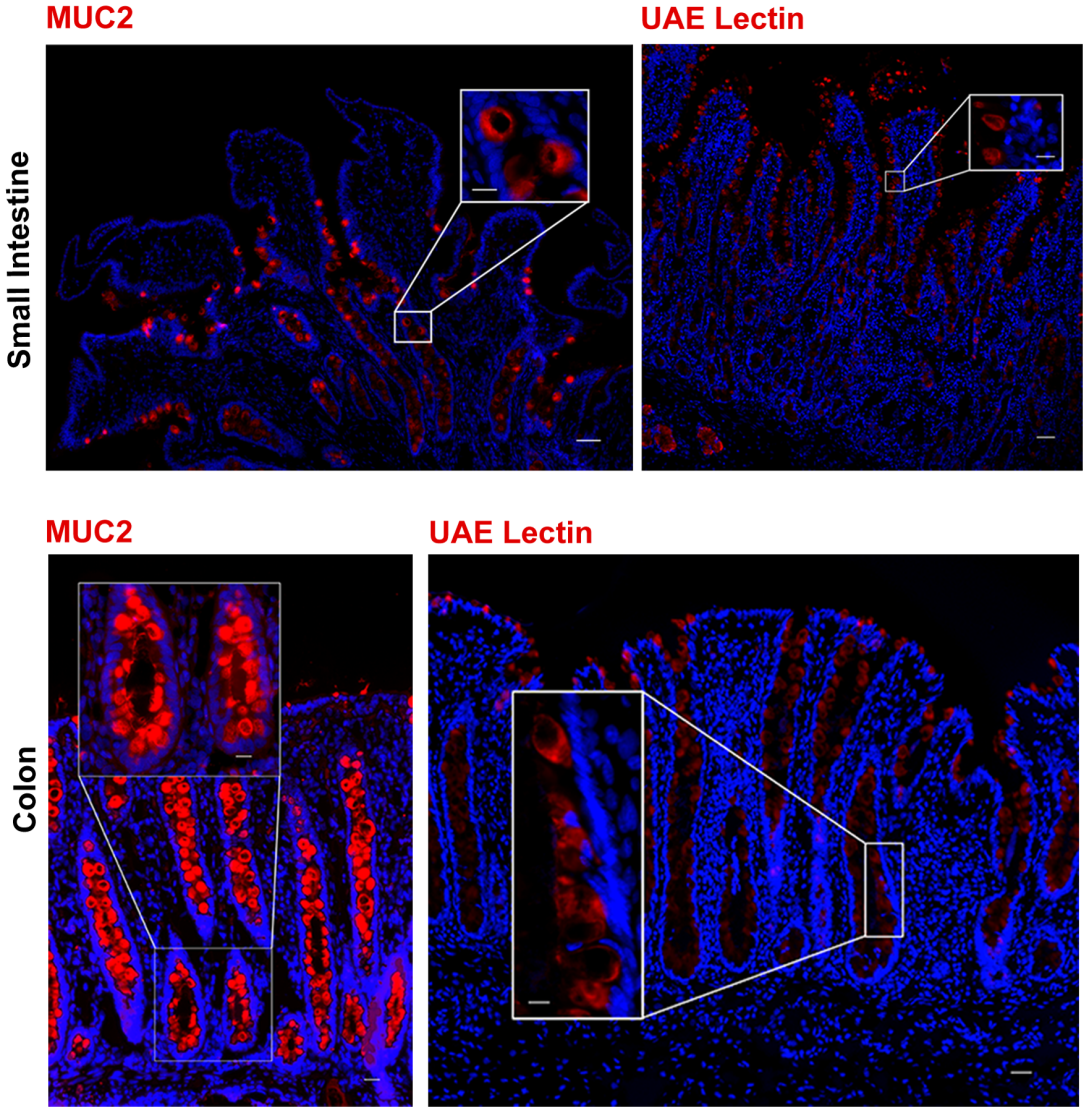
Markers to identify goblet cells. Identification of goblet cells in porcine small intestine and colon are shown. Goblet cells, the mucus producing cells, were identified by immunostaining for MUC2, a component of mucin, and UEA-1, a lectin that specifically binds to alpha-linked fructose receptors. The mucinous secretions of multiple cells in both the small intestine and colon were positively identified. All specific markers (red). Nuclei (blue). Scale bar 200 µm, inset scale bar 50 µm.

Secretory goblet cells produce mucins that are integral to intestinal physiology by providing protection of the epithelial surface as well as aiding in absorption [55]. Immunostaining for Mucin 2 (MUC2) exclusively marked mucous-producing cells along the entire length of small and large intestine as well as the crypt-villus axis (Figure 6). Positive staining was localized to the cytoplasm of positive cells and within the luminal surface which is the expected location of mucinous secretions. *Ulex europaeous* agglutinin-1 (UEA-1) is a lectin that specifically binds to alpha-linked fructose receptors located on cell surface glycoproteins and glycolipids and is used to detect both goblet and Paneth cells in mice [56]. UEA-1 bound to the mucinous secretions of goblet cells in porcine small and large intestine (Figure 6). Besides marking goblet cells in fixed tissue sections, UAE-1 is likely suitable for fluorescence activated cell sorting of live cells [37].

The existence of the Paneth cell in the pig remains disputed [57], [58]. No eosin or toluidine blue staining, which typically marks apically located granules in Paneth cells, was identified in the crypt base of porcine small intestine (data not shown). In mice lysozyme expression is a biomarker for Paneth cells [59], [60]. Lysozyme staining was not observed in the epithelium of porcine small intestine despite the use of multiple anti-lysozyme antibodies (data not shown; Table 2).

### TEM characterization of porcine crypt-based cells

**Figure 7:**
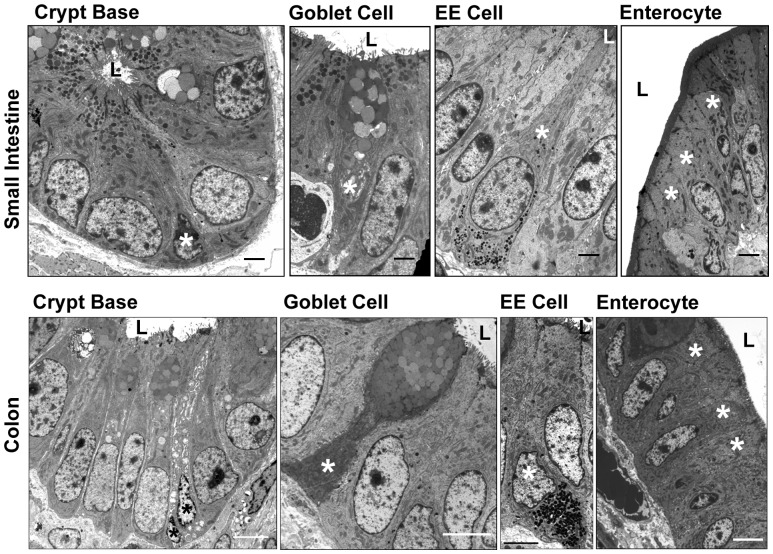
Transmission electron microscopic epithelial characterization. The ultrastructural appearance and characterization of porcine small intestine and colonic epithelial cells are shown. All cell types were morphologically distinguishable. Crypt base columnar stem cells, goblet cells, enteroendocrine cells, and absorptive enterocytes are all marked with asterisks. ‘L’ indicates lumen. Small Intestinal images, scale bar 2 µm. Colon images, scale bar 5 µm.

Transmission electron microscopy allows for the morphological identification of cell lineage by the presence or absence of sub-cellular features [6], [33], [61]–[63]. Comparative analysis of crypt-based cells from various organisms has enabled the characterization of cell types that are consistent with particular lineages [6], [33]. A complete description of the pig small intestine and colon, to the best of our knowledge, has not been previously described. At least two distinct cell types were distinguishable in the crypt base of the pig small intestine and colon (Figure 7). Multiple irregularly shaped, small, columnar cells with basally located nuclei and scarce cytoplasm, consistent in appearance with CBC cells of mice and humans [33], [64], were interspersed between large pyramidal shaped cells with large supranuclear clear mucoid vesicles and small electron dense bodies (Figure 7). The appearance of these large mucoid filled cells was not entirely consistent with the accepted morphological features of Paneth cells in other mammalian species [63], [65]. The goblet cell within the small intestine and colon of the pig possessed small basally located nuclei that were notably distended apically with mucinous globules consistent with the accepted ultrastructural appearance in mammalian intestinal tissue (Figure 7) [61], [66]. The enteroendocrine cells of the pig intestine demonstrated a narrow apex and wide base with many small, spheroidal, electron dense granules in the infranuclear region as is classically described in mammals (Figure 7) [62], [66], [67]. The mature absorptive enterocytes of the pig intestine were clearly distinguishable as columnar shaped cells with centrally located nuclei. Other key features of these cells include multiple organelles and a lack of secretory granules within the cytoplasm and a position closer to the gut lumen in both the small intestine and colon, as has been described in other mammals (Figure 7) [33], [66]. These electron micrographs of the porcine crypt base represents a foundation for morphologic description of cells in normal, injury and disease states.

### Assessment of gene expression in stem/progenitor and differentiated cell lineages

Measuring gene expression is essential to understanding mechanisms of injury, disease and the stem cell-driven regeneration. Because qPCR primer sets to detect target gene expression in pig are limited, we designed and validated primers to genetic biomarkers of stem/progenitor cells, differentiated cell lineages, and important signal transduction pathways involved in regeneration [38], [44], [68], [69]. Twenty one candidate PCR primers were designed and six were previously described [27], [70], [71] (Table 3). Amplification of cDNA generated from total intestinal RNA demonstrated expression of Wnt3a^+^ and Lgr5^+^, which are important regulators of IESC maintenance [59], [59,72]. Genes important for Notch pathway regulation, Atoh1^+^, Dll4^+^, and Hes1^+^, were amplified from epithelial-derived cDNA. These genes are critical for appropriate cell differentiation and fate and important to interrogate in regeneration [38]. ‘Active’ stem cell markers, Olfm4^+^, Ascl2^+^, Sox9^+^, CD24^+^, were used to monitor stem cell renewal and maintenance. Hallmark genetic biomarkers for differentiated lineages were detected by amplification of Muc2^+^ and Itf^+^ (goblet cells), CgA^+^ and Cck^+^ (enteroendocrine cells), and Sglt1^+^ and L-Fabp^+^ (absorptive enterocytes) [43], [73]–[75]. Single amplicons of the appropriate size were verified by gel electrophoresis and DNA sequencing of all amplicons validated target gene amplification. Primers efficiencies were calculated using the equation, Efficiency  = 10∧(−1/slope) –1 and demonstrated >92% efficiency (Table 3). Under the conditions described, no primer-dimers were observed.

**Table 3:** Primers designed for gene expression in porcine intestine.

Target	Sequences of primers (5′ to 3′)	Annealing Temp(°C)			Product Size	Reference
House Keeping Genes						
Gapdh	ATCCTGGGCTACACTGAGGAC AAGTGGTCGTTGAGGGCAATG	60				
Gusb	TAACAAGCACGAGGATGCAG TCCTCTGCGTAGGGGTAGTG	60		129		Author
18S	TGGAGCGATTTGTCTGGTTA ACGCTGAGCCAGTCAGTGTA	60		200		[71]
IESC						
Lgr5	CCTTGGCCCTGAACAAAATA ATTTCTTTCCCAGGGAGTGG	60		110		Author
Olfm4	GTCAGCAAACCGGCTATTGT TGCCTTGGCCATAGGAAATA	60		226		Author
Sox9	CGGTTCGAGCAAGAATAAGC GTAATCCGGGTGGTCCTTCT	60		229		Author
Ascl2	GAGCTGCTCGACTTCTCCAG TTCCACACTAGCCCTTGGTC	60		204		Author
CD24	TAAGAGCCAGCGGTCCTCTA GACCGAGAGCACGAAGAGAC	62		283		Author
+4 Stem Cells/Quiescent						
Bmi1	TCATTGATGCCACAACCATT TGAAAAGCCCCGGAACTAAT	60		189		Author
Proliferative Cells						
Pcna	TACGCTAAGGGCAGAAGATAATGCTGAGATCTCGGCATATACGTG	60		192		[27]
Atoh	CACGGGCTGAACCACGCCTT GGTACCCGCGCTTGCTTCGT	60		234		Author
Hes1	ATTCCTCGTCCCCGGTGGCT TGCTTAGCGCGGCCGTCATC	62		279		Author
Goblet Cells						
Muc2	GGCTGCTCATTGAGAGGAGT ATGTTCCCGAACTCCAAGG	60		249		Author
Itf	TCGGTTCCCCAGAACCTGCCC CGGGGATGCTGGAGTCGAAGC	60		220		Author
Enteroendocrine Cells						
CgA	GACCTCGCTCTCCAAGGAGCCA TGTGCGCCTGGGCGTTTCTT		60	332		Author
Cck	CAAAAGGTAGACGGCGAGTC GCGGGGTCTTCTAGGAGGTA		60	217		Author
Enterocytes						
Sglt1	GCAGCTGTCTTCCTACTTGC GCAAACTCGGTAATCATACGG		60	113		
L-Fabp	CCGGCAAATACCAAGTACAGAGCCCCTTCTCCCCAGTCAGGGTCTCC		60	225		Author
Growth Factors and Signaling Molecules						
Tgfα	CAGCTGTGGTGTCCCATTTT TAATGGCCTGCTTCTTCTGG		62	189		Author
Egf	TGGCAGATGCTGGAATATCA AAGGCGCTTAAGAGAACACG		60	262		Author
Dll4	TCATCATCGAAGCTTGGCAC GCGCTTCTTGCATAGACGTG		60	224		Author
Wnt3a	GCGACTTCCTCAAGGACAAG GGTCACGTGTACCGAAGGAT		60	201		Author
Wnt11	CGTGTGCTATGGCATCAAGT TCTCTCCAGGTCAAGCAGGT		60	259		Author
Lyz	GGTCTATGATCGGTGCGAGT AACTGCTTTGGGTGTCTTGC		62	220		Author
pBD1	ACCGCCTCCTCCTTGTATTC GGAGCAGCTTCTGAGCCATA		60	233		Author
pBD2	ATGAGGGCCCTCTGCTTGCT ATACTTCACTTGGCCTGTGTGTCC		60	259		[70]
Apoptosis						
Casp3	ACCCAAACTTTTCATAATTCA ACCAGGTGCTGTAGAATATGC		60	143		[27]

### In vitro culture of porcine crypts

**Figure 8:**
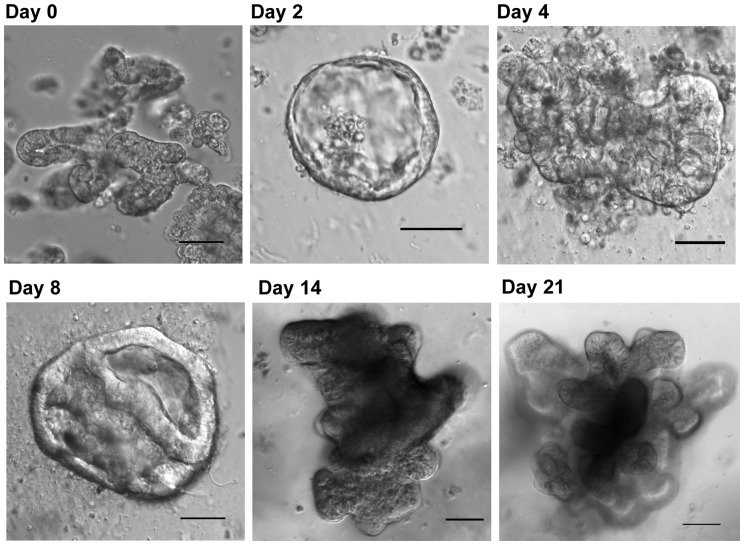
*In vitro* culture of porcine crypts. The *in vitro* isolation, growth and maintenance of enteroids derived from porcine intestinal crypts are shown. On the day of collection (day 0) crypts maintain their morphologic appearance. As the enteroids develop they become enterospheres (day 2) and progressively enlarge and form complex structures with a pseudolumen and crypt-like structures (days 4, 8, 14, 21). Scale Bar 100 µm.

**Figure 9:**
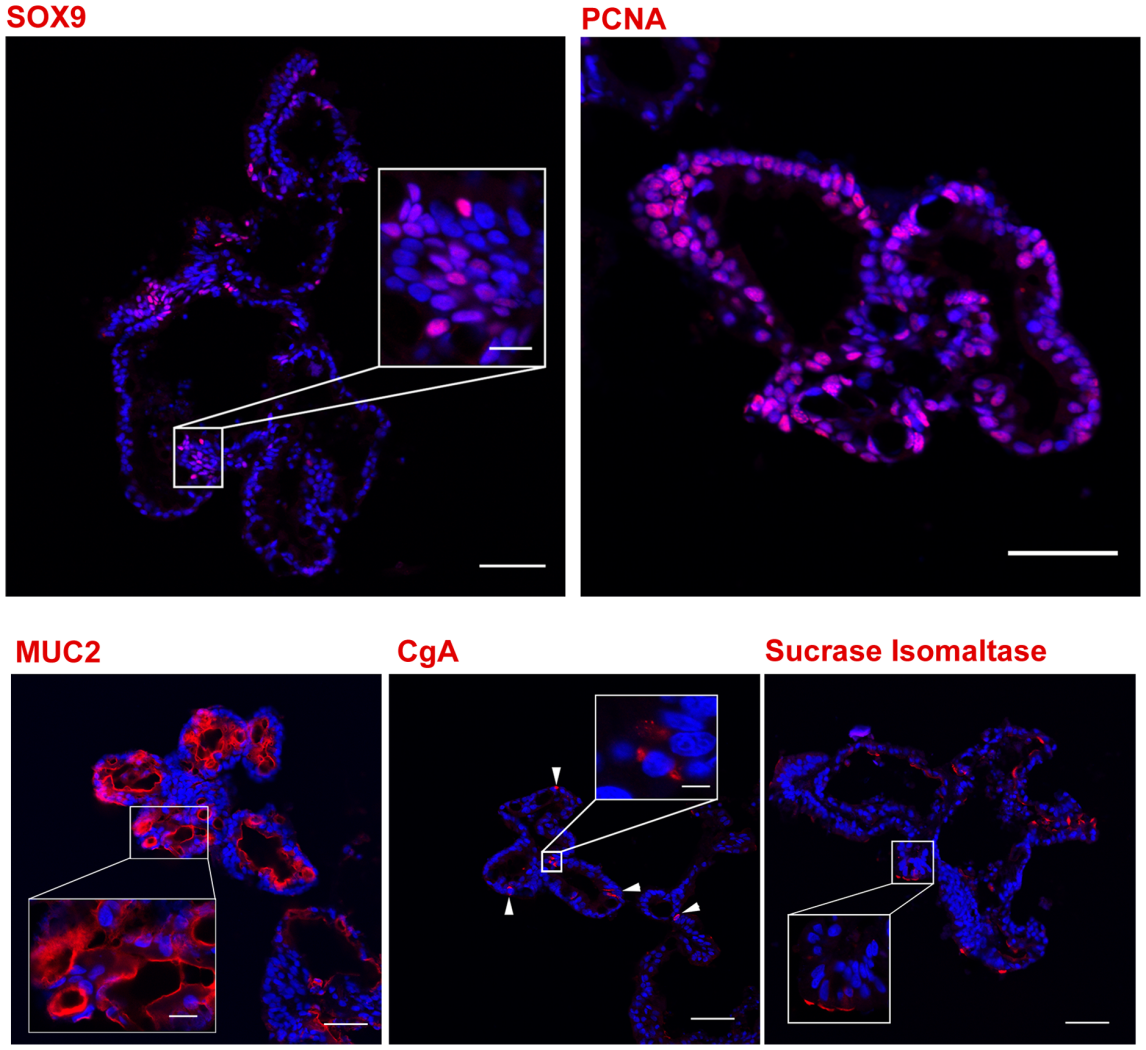
Markers to identify cell lineages within *in vitro* cultures. The identification of specific cell lineages within *in vitro* cultures of porcine crypts is shown. The existence of stem/progenitor and differentiated lineages were confirmed in enteroids utilizing the established genetic biomarkers for cell lineage identification: anti-SOX9 (stem/progenitor), anti-PCNA (proliferation), anti-CgA (enteroendocrine), anti-MUC2 (goblet) and anti-sucrase isomaltase (absorptive enterocyte) antibodies. All specific markers (red). Nuclei, blue. Scale Bar 50 µm, inset scale bar 10 µm.

Long-term culture of intestinal epithelial stem cells in mice and humans has only recently been accomplished and has revolutionized the ability to conduct detailed mechanistic studies in a highly controlled manner [38], [76]–[78]. Pig crypts isolated from jejunal tissue were introduced into a modified 3-dimensional (3-D) culture environment similar to the culture conditions that support growth of mouse and human enteroids [42], [76]–[79]. Within 24 hours of plating whole pig crypts in matrigel with defined medium, enterosheres formed (Figure 8, day 2) [80]. These structures persisted until day 4 and then began to convert into enteroids that possessed columnar epithelial cells and primitive crypt buds (Figure 8, day 4). Mature crypt buds developed by day 14 and by day 21 enteroids were fully formed (Figure 8, day 14 and 21). Enteroid cross sections demonstrated the presence of SOX9^+^ stem/progenitor cell populations, PCNA^+^ zones of proliferation, MUC2^+^ goblet cells, CgA^+^ enteroendocrine cells, and SIM^+^ absorptive enterocytes (Figure 9). Fully developed enteroids were allowed to persist in culture for two weeks at which point they were passaged. To date, enteroids have been passaged 8 times representing a total of 4.5 months in culture. There has been no apparent decrease in enteroid formation over this time.

## Discussion

A recent NIH symposium entitled “Improving Animal Models for Regenerative Medicine” focused on the development of large animal models for the study of human disease [81]. The motivation for the symposium was the persistent failure in translating murine models to clinical treatments [81]. The utility of the pig as a large animal model has been well-documented for many body systems [15]–[21], [82]–[88]; and the similarities between the pig and human gastrointestinal system position the pig as a promising species for animal models of gastrointestinal disease. Seminal advances made in murine intestinal stem cell biology now position investigators to answer clinically relevant problems from the perspective of stem cell-driven epithelial regeneration. Data presented in this study lay the foundation for developing the pig as a large and physiologically relevant animal model for these studies. This study identifies, develops and validates a range of genetic biomarkers and crypt culture strategies that will enable investigators to assess stem cell maintenance and potency in both the small and large intestine of pig models of physiology, injury and disease.

Few studies use commercially available antibodies on porcine intestinal tissue to assess cell lineage allocation, thus limiting the ability to effectively monitor and analyze epithelial regenerative responses [10], [25]–[27], [89], [90]. To address this problem, we identified a comprehensive set of commercially available antibodies that would cross-react with target pig proteins to enable detection of stem/progenitor and post-mitotic lineages. The ability to specifically observe epithelial dynamics during physiology and disease is critical to understanding and quantifying regenerative processes and therapeutic interventions [10]. Appropriate designation of EpCAM expressing cells, for example, can offer insight into epithelial cell-cell adhesion, migration, signaling, differentiation and proliferation since it plays a key role in these cellular functions [35]. Additionally, assessing the proliferative capacity of IESCs and their progenitors is essential for monitoring regenerative responses in the small intestine and colon. Detailed analysis of cell cycle progression during injury and disease states could yield important information pertaining to repair mechanisms. The maintenance of epithelial barrier function depends on the tightly controlled balance between cellular proliferation, differentiation and apoptosis. Monitoring the phenotypic changes resulting from injury or in genetically modified animals can shed light into critical homeostatic pathways. These pathways can now be monitored in pig models utilizing the biomarkers for PCNA, MCM2, BrdU, pH3 and CASP3.

Identification of stem and progenitor cell populations has proven critical to deciphering important cellular pathways as well as the impact and response of these cells to injury. In mice, ‘active’ IESCs are marked by *Lgr5, Olfm4, Ascl2, Sox9* [31], [43], [59], [60], [73], [74], [91], [92]. Unfortunately, of the commercially available antibodies for CBCs tested, only SOX9 demonstrated positive staining. *Sox9* is a member of the SRY-family of transcription factors that is primarily expressed in crypt-based columnar stem cells and transit-amplifying progenitor cells [31], [42], [43]. Enteroendocrine and Tuft cells also express very high levels of *Sox9* and recent evidence suggests that the *Sox9* high population has ‘reserve’ IESC capacity [31], [42]–[44], [75]. Slower dividing ‘reserve’ or facultative stem cells primarily reside above the Paneth cell compartment in mice and humans [41], [44]. These have been historically termed ‘the +4 stem cells’ which denotes the cell position in the crypt base where they most likely exist [93]. As the name suggests, facultative IESCs appear to respond to injury stimulus to re-enter an active state to regenerate the epithelium [44], [69], [94]. To some extent, the reserve IESC population is marked by *Bmi1, Tert, Hopx*, and *Lrig1* in mice [37], [95]–[101]. Immunostaining for HOPX will enable future studies utilizing porcine models to evaluate the role of these putative ‘reserve’ IECSs during and following intestinal injury.

The ability to observe and quantify fully differentiated cells is critical to understanding the dynamics of epithelial regeneration in normal homeostasis, injury, and repair. CgA was used as a general marker of enteroendocrine cells but multiple sub-types were also characterized. The hormones produced by all of the enteroendocrine cells are integral to crypt cell physiology. Proglucagon, for example, is produced and cleaved within the L-type enteroendocrine cells into glucagon-like peptide-1 (GLP-1) and glucagon-like peptide-2 (GLP-2) [54]. Interestingly, glucagon-like peptides demonstrate immunoreactivity to antibodies against glucagon, the product of cleaved proglucagon in the pancreas [54]. The potential therapeutic benefits of both GLP-1 and GLP-2 are of interest for multiple important human diseases. GLP-1 is integral to both signaling satiety and in glucose homeostasis. GLP-1 based treatments are now well established in the management of type 2 diabetes and have been proposed for the treatment of obesity [102]. Research into the therapeutic benefits of GLP-2 administration to hasten epithelial proliferation with direct stimulatory effects on the stem cell population following intestinal resection have also been studied [12], [103]–[105]. The ability to clearly identify GLP-1 and GLP-2 producing cells may then prove integral in pig models that study weight management, diabetes and short bowel syndrome [12], [102], [103], [106].

Electron microscopy supported the immunohistochemical findings on porcine small and large bowel showing clear ultrastructural characteristics of lineage states along the crypt villus axis. Electron microscopy provided morphological evidence suitable for identification of cells consistent with CBC stem cells, goblet cells, enteroendocrine cells and absorptive enterocytes. TEM studies have and will continue to contribute to understanding the ultrastructural cellular changes that occur during disease and repair processes as well as following signaling pathway manipulation that may not be possible with immunohistochemical studies [95], [107], [108]. TEM has been utilized, for example, to visualize invasion of intestinal epithelium by viral particles and detailed evidence of the impaired structural integrity of epithelial tight junctions in disease [108]–[110].

Quantitative gene expression analysis is highly sensitive and contributes to a more complete characterization of intestinal epithelium in the pig. In this study, the PCR primers were specifically designed to amplify target genes currently used as molecular signatures for each cell type and important signaling molecules known to regulate intestinal homeostasis. Interpreting gene expression dynamics during and following intestinal injury can give insight into the cell populations, CBCs (Lgr5^+^, Olfm4^+^, Sox9^+^, Ascl2^+^, CD24^+^) versus ‘reserve’ stem cells (Bmi1^+^), that are compromised or stimulated in the process [44], [69], [95]. Additionally, the impact on stem cell homeostasis (Wnt3a^+^), proliferative capacity (PCNA^+^), and cell fate determination (Atoh^+^, Hes1^+^) can be evaluated to interpret epithelial regenerative capacity [44], [69], [111]. Ultimately, by evaluating the specific post-mitotic cell populations, absorptive enterocyte (Sglt1^+^, L-Fabp^+^) or specific secretory cells (Muc2^+^, Itf^+^, CgA^+^, Cck^+^) that are sensitive to or upregulated in response to injury, potential therapeutics aimed at signal pathway manipulation can be pursued [38], [75], [112], [113]. Having defined qPCR primers allows for thorough and reliable interpretation of cellular pathway dynamics, and therefore, insight into mechanistic processes controlling epithelial regeneration.

An intriguing observation in cross species comparison of the small intestine epithelium is the presence of the Paneth cell lineage in some species and the absence in others. Paneth cells are long-lived post-mitotic cells that reside in the base of the small intestinal crypts in some species. In mice, Paneth cells have been implicated in serving as a ‘nurse’ cell for the CBC stem cells by secreting WNTs and presenting NOTCH-ligands [38], [59], [60]. In the colon, evidence indicates that a c-KIT^+^ MUC2^+^ cell may serve as an analogous counterpart to the Paneth cell in the colon [114]. The existence of Paneth cells in pigs is still debated [57], [58]. Morphologically, Paneth cells can be identified by the ultrastructural presence of an elongated flattened nucleus, large cytoplasm and secretory granules [63], [65]. In our porcine studies, TEM and immunostaining do not support the presence of a *bona fide* Paneth cell; however, our study presents data that is consistent with the interpretation that a Paneth cell equivalent, similar to that of the mouse colon, may be present in pig small intestinal crypts.

TEM and morphometric analyses presented in our study indicate that at least two different cell types reside within the crypt base. A small columnar shaped cell with a basally located nucleus and sparse cytoplasm seems consistent with the accepted ultrastructural appearance of the CBCs in other species. Interspersed between these cells is a larger cell type with electron dense supranuclear secretory vesicles. Many of these cells also contain clear, mucoid in appearance, apically located vesicles. Interestingly, the ultrastructural appearance of the secretory granules vary in size and number as well as electron density between those species accepted as having Paneth cells [65]. Post-mitotic cells (PCNA and MCM2 negative) are also present at the crypt base in the pig. These cells appear to be Sox9^+^ and Muc2^+^, and UEA-1^+^, which is consistent with the Paneth-like cells identified in mouse colonic crypts and marked by c-KIT [114]. Unfortunately, we were unable to verify c-KIT staining in these cells due to the lack of cross-reactivity with the c-KIT antibodies tested. These data point to the need for more detailed analysis of gene expression to determine if they produce functionally relevant mitogens and morphogens similar to Paneth or Paneth-like cells in the mouse small intestine and colon.

Besides enabling detailed mechanistic studies *in vitro*, long-term crypt culture from the pig small intestine represents a significant advancement toward developing tissue-engineering strategies and stem cell-based therapies. Organoid units from primary small intestine have been placed on biodegradable scaffold tubes and then implanted into the omentum of an autologous host [25]. The engineered tissue demonstrated morphological characteristics of small bowel 7-weeks post implantation. While these studies used biopsied samples derived from resected tissue, it is likely that a therapeutic mass of tissue would be required to be clinically relevant. The culture method developed in this study will enable a small number of crypts, perhaps from a biopsy, to be expanded *ex vivo* to increase the mass of epithelium required to test therapeutic strategies for tissue replacement. Detection of successful and functional tissue replacement is fundamental to monitoring outcomes of these new approaches to treat disease and injury to the intestinal epithelium. This new long-term crypt culture model also represents a significant advancement toward development of pharmaceutical screening modalities, stem cell therapy models, and tissue replacement strategies that can all be tested in a translationally relevant context. The comprehensive set of reagents identified, developed and validated will serve as a foundation for using the pig as a translational model to study stem cell-driven regeneration of the intestinal epithelium.
